# A picture of purpose: exploring veterinary students’ motivations through photo elicitation

**DOI:** 10.3389/fvets.2025.1553433

**Published:** 2025-03-24

**Authors:** Ronald J. Orchard, Cassidy Moreau, Brad Crauer

**Affiliations:** ^1^Department of Clinical Sciences, College of Veterinary Medicine, Kansas State University, Manhattan, KS, United States; ^2^Veterinary Health Center, College of Veterinary Medicine, Kansas State University, Manhattan, KS, United States

**Keywords:** veterinary education, photo elicitation, student motivation, thematic analysis, resilience, human-animal bond, spectrum of care, professional identity

## Abstract

Understanding the motivations that drive veterinary students is essential for supporting their academic success, wellbeing, and professional satisfaction. This qualitative study utilized photo elicitation, a visual and narrative research method, to explore the personal, emotional, and professional “whys” of 14 veterinary students pursuing a Doctor of Veterinary Medicine (DVM) degree. Participants submitted photographs and reflective narratives describing their motivations and how these motivations supported their journeys. Thematic analysis revealed five key themes: (1) Deep Emotional Bonds with Animals, (2) Overcoming Personal Adversity, (3) The Influence of Role Models, (4) Desire to Make a Difference, and (5) Community and Service. These findings highlight the central role of formative experiences, relationships, and values in shaping students’ aspirations and perseverance. The study underscores the potential for reflective practices, such as photo elicitation, to reconnect students with their intrinsic motivations, fostering resilience and long-term satisfaction in veterinary practice. Integrating principles such as compassionate advocacy, spectrum of care, and community engagement into veterinary education may better align training with students’ values, enhancing their preparation for impactful and fulfilling careers.

## Introduction

Veterinary education demands resilience, adaptability, and a deep sense of purpose—qualities that have been identified as key predictors of wellbeing and persistence in medical and veterinary professions. Resilience, the ability to recover from setbacks and maintain motivation under stress, is particularly crucial for veterinary students, who often face emotional and academic challenges (1). Similarly, a clear sense of purpose has been shown to enhance persistence and professional fulfillment, reinforcing students’ ability to navigate difficulties and maintain long-term engagement in their careers (2, 3). Gasper (4) further emphasizes that resilient individuals in medical fields exhibit adaptability, resourcefulness, and self-directedness, allowing them to thrive despite uncertainty. While other attributes such as integrity and honesty are undoubtedly essential in veterinary practice, our study focuses on these three qualities because of their documented impact on student success and professional longevity.

Despite its rigorous training, veterinary education often prioritizes technical competencies over the cultivation of reflective practice, personal meaning-making, and adaptability (5). Yet, fostering intrinsic motivation and self-awareness may help counteract known challenges in the field, such as high rates of burnout, mental health struggles, and attrition (6, 7). Research suggests that students who engage in reflective practices early in their careers are better equipped to sustain their professional identities and manage occupational stress (1). While veterinary curricula effectively equip students with the medical and technical knowledge required for practice, they often lack dedicated strategies to address the psychological resilience and adaptability necessary for career sustainability.

This study leverages photo elicitation, a qualitative research method that integrates visual and narrative elements to explore veterinary students’ motivations. This method has been widely used in education research to elicit personal meaning, foster engagement, and deepen insight into participants’ lived experiences (8). It is particularly valuable in professional identity studies, where verbal responses alone may not fully capture the complexity of motivations and personal reflections (9). Previous studies have successfully applied photo elicitation in human medical education, where it has been shown to enhance self-awareness, professional identity development, and emotional processing (10, 11). However, photo elicitation remains largely underutilized in veterinary education, with only one study these researchers could find incorporating visual methodologies to investigate veterinary students’ perspectives (5). One goal of this study is to continue to introduce veterinary educators to this methodological approach, demonstrating its potential to enrich understanding of student experiences and motivations.

Photo elicitation is sometimes conflated with photovoice, a participatory action research method that engages participants as documentarians, commentators, and potential agents of social change (11). The two terms are often used interchangeably, but photo elicitation focuses on the interview process itself, whereas photovoice involves a broader participatory and action-oriented framework. As Bugos et al. (11) explain, “Photo-elicitation is a core component of photovoice, which was first described in 1997 and is a form of community-based participatory research that engages participants at each step of the research process as documentarians, commentators, and agents of social and political change.” In this study, students took or shared their own photos rather than responding to researcher-provided images, which aligns with aspects of photovoice. However, given that the primary aim was to explore student perspectives rather than facilitate a structured action-oriented intervention, we follow Bugos et al. (11) in using the term photo elicitation to describe our approach.

The guiding question for this study was: *What are the motivations (“why”) of veterinary students for pursuing a Doctor of Veterinary Medicine (DVM) degree, as expressed through photo elicitation and accompanying narratives?* By inviting participants to select or create photographs representing their “why” for pursuing a DVM, this approach provided a rich lens into the values and experiences shaping their journeys. The integration of visual imagery enabled participants to articulate dimensions of their motivations that might remain inaccessible through verbal inquiry alone.

Five key themes were constructed from this dataset: (1) Deep Emotional Bonds with Animals, (2) Overcoming Personal Adversity, (3) The Influence of Role Models, (4) Desire to Make a Difference, and (5) Community and Service. These themes underscore the deeply personal and relational aspects of veterinary students’ motivations while also reflecting broader principles such as spectrum of care (SoC), community engagement, and reflective practice. By examining these themes, this study not only illuminates the factors sustaining veterinary students but also highlights opportunities for enhancing veterinary education in ways that support both student wellbeing and professional competency.

As a preventive approach, this study explores how early engagement with students’ intrinsic motivations—their “why” for pursuing veterinary medicine—may serve as a protective factor, fostering resilience and professional fulfillment. Rather than suggesting that education should be dictated by student preferences, we argue that integrating reflective practices alongside rigorous clinical training can help students develop a deeper connection to their purpose, enhancing their ability to navigate the inevitable hardships of the profession. Given the well-documented challenges of veterinary mental health, incorporating resilience-building strategies from the outset of training is not simply a pedagogical preference but an ethical necessity.

## Methods

### Photo elicitation as a method

Photo elicitation combines participant-selected images with reflective narratives to explore participants’ perspectives, emotions, and lived experiences. Originally introduced by Collier (12) in anthropology, this method has been widely adopted in educational and healthcare research due to its ability to elicit deeper emotional and cognitive engagement than verbal-only approaches (9, 11). Photo elicitation has been successfully used in medical education to explore student identity, professional motivation, and emotional resilience (13, 14), but its use in veterinary education remains limited to a single published study (5). Given the personal and emotionally charged motivations behind veterinary students’ career choices, photo elicitation was well-suited to uncover nuanced perspectives that might remain unspoken in traditional interviews (10).

The method used in this study aligns with the principles of photovoice, a form of community-based participatory research that allows participants to document and interpret their lived experiences through photography (11). However, while photovoice often emphasizes action-oriented social change, this study primarily employed photo elicitation as a tool for exploring students’ personal and professional motivations. By inviting participants to select or create photographs representing their “why” for pursuing a DVM, this approach provided a rich lens into the values and experiences shaping their journeys. The integration of visual imagery enabled participants to articulate dimensions of their motivations that might remain inaccessible through verbal inquiry alone, reinforcing the value of multimodal reflection in veterinary education (8).

This method is particularly relevant for veterinary education research, where students’ motivations are deeply rooted in personal relationships with animals, formative life events, and a sense of professional calling. The visual-narrative format allows researchers to bridge the gap between abstract constructs, such as resilience and purpose, and the tangible, lived realities of students’ academic journeys (13, 14). While resilience and purpose are often discussed in veterinary education as conceptual goals, their development is deeply personal and shaped by individual experiences. Traditional qualitative methods, such as interviews or surveys, may struggle to capture the emotional and symbolic dimensions of these motivations.

Photo elicitation, by incorporating participant-selected imagery, allows for a richer, more layered representation of these concepts (9, 11). The inclusion of visual elements provides a concrete reference point for students’ reflections, making their narratives more detailed and emotionally resonant (15, 16). Prior research in education has demonstrated that photo elicitation fosters deep self-reflection and perspective shifts, enabling students to articulate experiences with greater nuance than in text-based interviews alone (10). Studies in medical education further indicate that this method enhances students’ ability to construct meaning from their experiences, fostering a more holistic understanding of professional identity and personal growth (13, 14). In this way, photo elicitation serves as a methodological bridge, translating complex, subjective experiences into analyzable qualitative data while maintaining the depth and authenticity of participants’ lived experiences.

Veterinary educationalists benefit from understanding students’ experiences and motivations from their own perspectives. Photo elicitation provides a unique lens to view the world as participants experience it, fostering empathy and uncovering the layers of meaning that underpin their professional aspirations (10). By inviting participants to select or create photographs representing their “why” for pursuing a DVM, this study encouraged them to reflect on their intrinsic motivations and how these motivations support their persistence through the academic challenges of veterinary training.

The participatory nature of photo elicitation empowered students to direct the research process by choosing images that held personal significance. This approach aligned with the goals of class, which emphasizes reflection, interdisciplinary collaboration, and the human-animal bond. Students’ narratives, combined with their photographs, provided researchers with holistic insights into the emotional and symbolic dimensions of their motivations, extending beyond what verbal responses alone could reveal (15).

### Participants and context

Fourteen veterinary students participated in this study, completing a demographic survey along with submitting photographs and written responses to the two questions below. These self-selected volunteers were enrolled in a pre-clinical elective course within the veterinary curriculum. The course focuses on equipping students with the skills to address access barriers in veterinary care, foster interdisciplinary collaboration, and strengthen the human-animal bond. Through lectures, practical experiences, and reflective assignments, the course emphasizes compassion and engagement, making it an ideal context for this research.

The impetus for this study emerged from an in-class assignment, initially designed as a reflective peer discussion activity. Students were asked to bring a personally meaningful photo that represented their “why” for pursuing veterinary medicine. In pairs, students shared their images, described their motivations, and were interviewed by a classmate before switching roles. The resulting conversations were notably rich and insightful, highlighting the potential of photo elicitation to uncover deeper narratives about professional identity and motivation. Given the depth of discussion and student engagement observed during the assignment, the method described in this paper was developed to systematically capture and analyze these perspectives in a research context.

While the study was conducted within a single elective course, it is important to consider how these students may compare to their peers who did not enroll in the class. Given the course’s emphasis on community engagement and access to care, it is possible that students who selected this elective were already more inclined toward reflective practice, service-oriented work, or non-traditional approaches to veterinary medicine. However, as veterinary students across diverse settings frequently express similar motivations—such as a deep connection to animals, overcoming adversity, or a desire to make a difference—the findings of this study likely reflect broader themes relevant to veterinary education as a whole. Future research could explore whether students outside this elective, or at different points in their training, articulate their motivations in comparable ways.

Table 1 highlights the demographic characteristics of the 14 participants.

**Table 1:** Participant demographic survey.

Category	Subcategory	Number of participants (*n* = 14)
Gender	Female	12
	Male	1
	Non-binary	1
First generation college student	Yes	1
	No	13
Upbringing	Rural	7
	Suburban	6
	Urban	1
Race/Ethnicity	White	14

In qualitative research, the logic of case selection differs fundamentally from that of quantitative sampling. Rather than seeking statistical generalizability, qualitative studies prioritize depth over breadth by selecting information-rich cases that offer meaningful insights into the phenomenon under investigation (17). Our sample size of 14 students aligns with established qualitative principles, wherein cases are selected for theoretical and conceptual significance rather than numerical representation (18, 19).

Moreover, this study follows a relational ethnographic approach (20), in which the emphasis is placed on how participants’ experiences and identities are socially constructed through their interactions with others and their professional environment. This perspective is particularly relevant to veterinary education, where students’ motivations and resilience strategies are shaped within a community of practice. As such, our findings do not claim universal applicability but instead offer theoretically transferable insights that contribute to broader discussions on student engagement, professional identity formation, and wellbeing.

Furthermore, our study reached thematic saturation, meaning additional data collection would have yielded diminishing new insights (17). The goal of qualitative research is not to count instances of themes but to develop a nuanced, contextual understanding of participants’ lived experiences (18). As Spillman notes, “qualitative research gains its explanatory power not through statistical inference but through careful, context-sensitive analysis of meaning and interaction” (18, p. 5). By focusing on a small but diverse group of veterinary students within a shared educational context, this study provides rich, theoretically grounded insights into the motivations and challenges of future veterinarians.

These methodological choices align with calls for qualitative veterinary education research to prioritize depth, reflexivity, and engagement with student perspectives rather than relying solely on large-scale surveys or quantitative metrics. Our approach contributes to an emerging body of literature that underscores the value of student narratives in shaping more resilient, ethically engaged, and community-oriented veterinary professionals.

### Implementation

Participants were asked to submit a photograph that represented their motivation for pursuing a DVM and to provide written reflections addressing two prompts:

1. Why did you choose this photo to represent your “why” for pursuing your DVM?
2. How has this motivation supported you during your academic journey in veterinary medicine?

Ethical guidelines for content submission were clearly communicated to participants, and all submissions adhered to these guidelines. This study received Institutional Review Board (IRB) approval from Kansas State University, ensuring ethical oversight of participant recruitment, data collection, and analysis. All participants provided informed consent and retained the right to withdraw from the study at any time without penalty. Data were anonymized and securely stored to protect confidentiality.

### Data analysis

**Figure 1:**
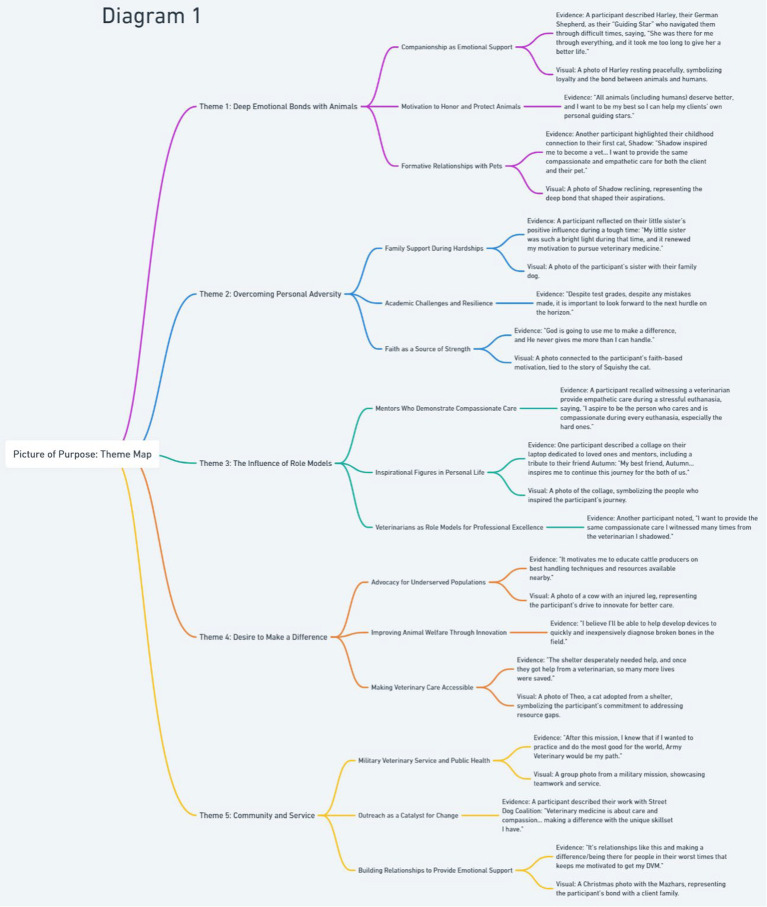
Illustrates the five overarching motivations identified in this population’s pursuit of a DVM degree, highlighting their connections to personal and professional experiences. The themes are supported by sub-themes that reflect participants’ stories and visual narratives. Each theme emphasizes the diverse ways students find purpose, resilience, and inspiration through relationships, mentorship, innovation, and service within the field of veterinary medicine.

The analysis followed the principles of reflexive thematic analysis (21, 22), which emphasizes the active role of researchers in constructing meaning rather than treating themes as passively emerging from data. Rooted in a constructivist epistemology, this approach recognizes that meaning is co-constructed through interpretation. The analysis was primarily inductive, guided by the data rather than predetermined categories, but remained theoretically flexible in considering broader concepts related to veterinary student motivations and professional identity formation. The following steps were undertaken:

1. Familiarization with data: the first phase involved deep engagement with the dataset. Researchers read and reread participant narratives alongside their chosen photographs, taking reflexive notes to capture initial impressions and areas of potential thematic significance. This step recognized that both textual and visual data contributed to meaning-making.
2. Generating initial codes: the textual narratives were systematically analyzed through open coding, with codes generated inductively from the data rather than determined *a priori*. Coding focused on capturing key ideas, emotional reflections, and conceptual markers related to students’ motivations. Given the multimodal nature of the data, photographs were analyzed for symbolic, contextual, and emotional significance, ensuring they were integrated into—not separated from—the interpretation process (22).
3. Developing sub-themes: coded data were examined for conceptual connections, leading to the identification of sub-themes, which represent patterns of meaning within the dataset. In line with Braun and Clarke (21), sub-themes function as intermediary analytic categories, highlighting nuances within broader themes. For example, within the overarching theme of *Resilience Through Connection*, sub-themes such as *Mentorship*, *Familial Support*, and *Faith as Motivation* captured distinct yet interrelated aspects of how students sustain their perseverance in veterinary school.
4. Constructing and refining themes: sub-themes were grouped into overarching themes that encapsulated the deeper narratives embedded within participants’ responses. This process was iterative, requiring continual movement between data, codes, and developing themes to ensure coherence and conceptual clarity (22). To visually organize and refine the relationships between sub-themes and themes, theme mapping was employed during this phase (as shown in Figure 1). This method facilitated a structured examination of how different aspects of students’ motivations interrelate, ensuring that themes were distinct yet interconnected.
  Additionally, researchers returned to the dataset after the initial theme development phase, checking for coherency, completeness, and fit of themes. Theme mapping also played a role in this verification process by providing a visual representation of analytical decisions, making it easier to identify overlaps, refine distinctions, and ensure alignment between themes and sub-themes.
5. Reviewing, defining, and validating themes: themes were further refined to ensure they were internally coherent (i.e., sub-themes meaningfully cohere under their parent theme) and externally distinctive (i.e., themes captured different aspects of the data rather than overlapping). Definitions were developed for each theme, clarifying their conceptual boundaries and ensuring they reflected the richness of participant narratives. Representative quotes were selected to illustrate each theme, demonstrating their interpretative depth.
  Additionally, to enhance trustworthiness and credibility, a member-checking process was conducted, in which participants were invited to review preliminary findings and provide feedback on whether the identified themes accurately captured their experiences. This process reinforced the validity of the analysis and ensured that the themes were grounded in participants’ perspectives.
6. Synthesizing and structuring results: the final themes and sub-themes were organized into a coherent analytical narrative, moving beyond description to interpretation. This phase emphasized how students’ motivations connect to larger professional, educational, and wellbeing considerations. The integration of visual data was critical in this process, as photographs often provided additional layers of meaning that might not have been apparent in textual narratives alone. For example, a participant’s image of their laptop covered in a collage of influential figures visually reinforced the interconnected nature of their motivations, adding interpretative depth to their written explanation.

### Reflexivity

This research is informed by a constructivist epistemology, recognizing that meaning is co-constructed through personal narratives, shared experiences, and reflection. Rather than assuming veterinary students’ motivations exist as fixed entities to be uncovered, we approached the data with the understanding that their professional identities are continuously shaped through interaction with people, animals, and educational environments. Our methodological choices—particularly the use of photo elicitation—were grounded in phenomenological and narrative identity perspectives, emphasizing how individuals assign meaning to experiences and construct their professional selves through storytelling (23, 24).

At the same time, critical theory (25) provided a framework for interrogating traditional power dynamics within veterinary education. Veterinary training has historically positioned the veterinarian as the ultimate authority, both in academic settings and in client-practitioner interactions. This study assumes that fostering reflective practice—particularly through multimodal approaches like photo elicitation—can help disrupt hierarchical learning models and encourage students to engage in collaborative decision-making, a key principle of Spectrum of Care (SoC) (26). In this way, we viewed students not just as subjects of a curriculum but as active participants in shaping their own professional development.

Our analysis was further guided by psychological resilience frameworks, which suggest that individuals develop adaptive capacities in response to adversity (27). As both researchers have professional experience in veterinary education—one as a veterinary social worker specializing in mental health and wellbeing, and the other as a veterinary educator with experience in clinical practice—we approached this study with an awareness of the significant stressors faced by veterinary students. This positioned us to interpret students’ narratives through the lens of resilience-building, considering how personal experiences may shape long-term professional fulfillment (2).

### Positionality of the researchers

The first author is a veterinary educator and PhD student in Leadership Studies, with a research focus on improving the Veterinary-Client-Patient Relationship (VCPR) through principles of community engagement and client-centered care. As a Mexican-American, non-traditional veterinary student who entered veterinary school after more than 15 years of veterinary experience, he witnessed firsthand the resilience strategies that helped practitioners sustain fulfilling careers. However, he also recognized that many veterinary students, particularly those entering directly from undergraduate programs, lacked the real-world experience necessary to contextualize their training and develop the psychological endurance needed for professional longevity. His scholarly motivation stems from a belief that all clients are worthy of a VCPR, and that veterinary education should integrate social science perspectives to better equip students for client communication, ethical decision-making, and navigating diverse care environments.

The second author is a veterinary social worker and mental health advocate, dedicated to supporting veterinary students and professionals through the emotional and ethical challenges of the profession. As the first veterinary social worker at Kansas State University, she has worked closely with veterinary educators and practitioners to develop mental health initiatives, facilitate community outreach courses, and promote interdisciplinary collaboration within veterinary medicine. Her work is guided by a belief in the transformative potential of the human-animal bond, and she approaches this research from a constructivist lens, recognizing that resilience, adaptability, and sense of purpose are co-constructed through personal narratives and shared experiences. She is committed to fostering inclusivity and belonging in veterinary education, acknowledging the systemic barriers that impact student wellbeing and professional identity development.

## Results

Through the analysis of participants’ photo submissions and accompanying narratives, five key themes identified that encapsulate the diverse motivations and inspirations behind veterinary students’ pursuit of a DVM degree. These themes comprise: (1) Deep Emotional Bonds with Animals, (2) Overcoming Personal Adversity, (3) The Influence of Role Models, (4) Desire to Make a Difference, and (5) Community and Service. Each theme is presented with a representative visual example and a corresponding narrative, illustrating the unique experiences and perspectives of participants.

Table 2 provides an overview of the key themes identified through thematic analysis of veterinary students’ photo and narrative submissions. Each theme is defined and accompanied by representative quotes that illustrate participants’ motivations for pursuing a DVM degree and the resilience strategies that sustain them throughout their academic journeys.

**Table 2:** Summary of themes with representative quotes.

Theme	Definition	Representative quotes
Deep emotional bonds with animals	Commitment to improving animal welfare and recognizing the profound human-animal bond.	*“Harley… was my Guiding Star. All animals (including humans) deserve better.”*
Overcoming personal adversity	Drawing strength from personal challenges, relationships, and resilience.	*“My little sister… renewed my motivation to pursue veterinary medicine. I applied that year and got in!”*
The influence of role models	Finding inspiration in mentors, family, and loved ones who provide guidance and motivation.	*“This photo includes everybody in my life who has ever motivated me to accomplish my dreams… My best friend, Autumn, inspires me to continue this journey for the both of us.”*
Desire to make a difference	Motivation to address challenges in veterinary care and improve animal outcomes.	*“An accident occurred while moving cattle… It motivates me to educate producers on best handling techniques and develop technology to diagnose injuries quickly in the field…”*
Community and service	Fulfillment through teamwork, outreach, and broader positive impact.	*“I love Army Vet Med… After this mission, I knew that if I wanted to practice and do the most good for the world, Army Veterinary would be my path to it.”*

### Theme 1: deep emotional bonds with animals

The most prominent theme highlighted the powerful emotional connections students share with animals, both their own pets and animals they have encountered through their lives. Participants described how animals served as sources of comfort, inspiration, and purpose. These bonds often formed the initial spark for their desire to pursue veterinary medicine.

- *Visual example*: A participant submitted a photo of Harley, their German Shepherd, peacefully resting on a bed. The image symbolizes the guiding role Harley played during the participant’s youth and the motivation to give animals a better life.
- *Accompanying narrative*: *“Harley was my Guiding Star and navigated me through the darkness of youth. She was there for me through everything, and it took me too long to give her a better life. All animals (including humans) deserve better, and I want to be my best so I can help my clients’ own personal guiding stars.”*

### Theme 2: overcoming personal adversity

Many participants shared experiences of personal hardship, ranging from challenging upbringings to struggles with self-doubt and mental health. Their perseverance and the support of animals or loved ones became driving forces in their pursuit of veterinary medicine.

- *Visual example*: A participant submitted a photo of their younger sister smiling next to the family dog. This image reflects the participant’s journey of renewal and motivation, driven by their sister’s presence during a difficult time.
- *Accompanying narrative*: *“I had a difficult time in undergrad due to some personal issues, and I eventually moved back home. My little sister was such a bright light during that time, and it renewed my motivation to pursue veterinary medicine. I applied that year and got in!”*

### Theme 3: the influence of role models

Role models, including family members, mentors, and friends, were cited as significant sources of inspiration. For some participants, these role models were individuals who modeled compassion and perseverance, while for others, role models were figures who supported and believed in them during their educational journey.

- *Visual example*: A participant shared a photo of a collage printed on a laptop sticker, dedicated to loved ones and mentors who motivated them to pursue their DVM. This includes a tribute to a close friend who tragically passed away while pursuing veterinary school.
- *Accompanying narrative*: *“This photo includes everybody in my life who has ever motivated me to accomplish my dreams… My ‘why’ today focuses significantly more on my passion for the people who love these animals… My best friend, Autumn, who showed me all the love and light, inspires me to continue this journey for the both of us.”*

### Theme 4: desire to make a difference

Participants expressed a strong desire to make meaningful contributions to animal welfare and to help those in need, particularly in underserved areas or through advancing veterinary care. Their motivations often stemmed from witnessing a lack of resources or the need for better care.

- *Visual example*: A participant submitted a photo of a cow’s leg injury in a field of harvested crops. This image represents their firsthand experiences with large animals and the need for better technology and support in rural areas.
- *Accompanying narrative*: *“An accident occurred while moving cattle, and by the time we arranged for a vet, the situation worsened. It motivates me to educate producers on best handling techniques and to develop technology to diagnose injuries quickly in the field without the need for travel.”*

### Theme 5: community and service

The final theme emphasized participants’ commitment to serving their communities, whether through public health initiatives, military service, or outreach efforts. These participants viewed veterinary medicine as a platform for engaging with their communities and creating a broader positive impact.

- *Visual example*: A participant submitted a photo of themselves and a group of Army veterinary professionals standing in front of a red tent with a “Paws and Claws” banner during a community outreach mission. This image highlights the participant’s dedication to military veterinary care and public service.
- *Narrative*: *“I love Army Vet Med and all of the good that they bring to the military as a whole. They allow our MWDs to be utilized, soldiers to keep pets on base, and food safety to be surveyed and confirmed. After this mission, I knew that if I wanted to practice and do the most good for the world, Army Veterinary would be my path to it.”*

## Discussion

The findings suggest that understanding students’ intrinsic motivations is not just an academic exercise but a critical component of fostering resilience, professional satisfaction, and ethical practice in veterinary medicine. Research has demonstrated that a strong sense of purpose predicts resilience and persistence in challenging academic and professional environments (2). However, veterinary education remains predominantly structured around hierarchical training models, often prioritizing technical expertise over student-driven meaning-making (25). Encouraging self-reflection through methodologies like photo elicitation may not only support student wellbeing but also cultivate collaborative problem-solving skills, which challenge rigid, top-down models of veterinary authority (1, 28).

One of the most pressing tensions in veterinary education is the balance between authoritative expertise and participatory, client-centered care. Historically, veterinarians have been positioned as the ultimate decision-makers in the exam room, an approach reinforced by traditional training methods that emphasize technical mastery over shared decision-making. This model is increasingly at odds with the spectrum of care (SoC) framework, which promotes collaboration between veterinarians and clients to develop contextually appropriate treatment plans (26). Research suggests that a lack of autonomy and flexibility in training contributes to stress and dissatisfaction in early-career veterinarians (1, 2, 28, 29). Without intentional efforts to foster adaptability and reflective practice, students risk entering clinical settings with rigid, one-size-fits-all approaches to patient care.

By integrating reflective exercises early in veterinary training, educators may foster a professional mindset that values contextualized, client-centered care rather than reinforcing outdated models of veterinary paternalism (29). Photo elicitation, as demonstrated in this study, offers a model for how veterinary education can cultivate these skills. This study highlights how photo elicitation provided deeper insights into veterinary students’ motivations compared to traditional qualitative methods. Previous research has shown that visual methodologies enable participants to access subconscious or difficult-to-verbalize emotions, fostering richer storytelling and engagement (9, 30). The inclusion of participant-selected images allowed for an additional layer of meaning-making, where the visual and textual elements reinforced and sometimes expanded upon one another.

For example, one participant’s image of their military veterinary team visually reinforced their emphasis on collaboration and service. Without this image, the written narrative alone might have suggested a more individualistic motivation rather than a collective professional identity. Similarly, a participant’s laptop collage symbolized generational resilience and legacy, themes that might have been less explicit without the visual representation. These findings reinforce the value of multimodal reflection in veterinary education, aligning with calls for participatory and experiential learning models that increase student engagement, resilience, and long-term job satisfaction (3, 31).

Beyond individual motivation, these findings align with broader concerns about wellbeing, job satisfaction, and retention in the veterinary profession. The 2023 Merck Animal Health Veterinary Wellbeing Study (28) reveals that veterinarians’ job satisfaction increases with age, particularly among individuals over 45, where “very/extremely satisfied” ratings are highest. However, veterinarians under 34 years of age report the greatest proportions of “somewhat” and “not too satisfied” ratings (29). These findings highlight a key opportunity for veterinary education: engaging students with their “why” early and intentionally. Creating spaces for students to articulate and reconnect with their motivations, such as reflective practices or experiential learning opportunities, may foster a stronger sense of purpose and mitigate early-career dissatisfaction.

By fostering environments that cultivate resilience, reinforce purpose, and encourage meaningful engagement, veterinary curricula can better align with the needs of students while upholding the profession’s core responsibilities. The five key themes identified in this study offer valuable insights into principles that can be integrated into veterinary education:

- Compassionate advocacy and spectrum of care

Implementing SoC principles helps future veterinarians deliver impactful, accessible care while reducing stress associated with rigid medical expectations (26, 32). The ability to navigate contextualized decision-making supports both professional fulfillment and ethical patient care (33–35).

- Resilience through reflective practice

Reflective practices, such as photo elicitation, create meaningful opportunities for students to articulate their personal motivations. Prior studies have shown that such reflection enhances resilience, self-awareness, and emotional wellbeing (13, 36, 37).

- Engagement and community service

Service-driven initiatives allow students to build technical, leadership, and interpersonal skills while directly addressing societal needs. Integrating veterinary community engagement (VCE) frameworks, outreach programs, and interdisciplinary collaborations into curricula reinforces the societal role of veterinary medicine and prepares students for complex, real-world challenges (38).

Participants’ motivations for compassionate advocacy, innovation, and service directly align with professional trends emphasizing community impact, ethical practice, and veterinarian wellbeing. Addressing student motivations early may foster job satisfaction as their careers progress. Notably, a Merck Study (28) found that veterinarians who perceive their work as meaningful report higher satisfaction, reinforcing the importance of cultivating purpose-driven engagement throughout veterinary education.

Moreover, principles such as spectrum of care and community engagement provide actionable frameworks for addressing challenges like burnout and access disparities. For example, adopting SoC practices can help veterinarians deliver equitable care without compromising their professional or emotional wellbeing (32, 35). Similarly, emphasizing collaboration and leadership in service-driven initiatives can strengthen the profession’s capacity to address pressing public health and animal welfare needs.

## Limitations

This study’s small sample size (*n* = 14) and lack of demographic diversity limit the generalizability of its findings. However, qualitative research does not aim for statistical generalizability but rather theoretical transferability, offering in-depth insights into participants’ lived experiences (17–19). Additionally, while all participants identified as White, this demographic homogeneity is not unique to our study but instead reflects the broader lack of diversity within veterinary medicine and veterinary education (39). The profession has long struggled with issues of racial and socioeconomic representation, and these systemic barriers inevitably shape the experiences and motivations of students entering the field. Future studies should actively seek to include more diverse perspectives to ensure a more comprehensive understanding of student motivations and identity formation.

The study was conducted within a single elective course, and students who enrolled may have already been inclined toward reflective practice, service-oriented work, or interdisciplinary perspectives. This may have shaped the types of motivations expressed and should be considered when interpreting the results.

The use of photo elicitation as a primary method introduces additional considerations. While this approach facilitated rich narratives, it required participants to actively select and interpret their own visual representations of motivation. This process may privilege individuals who are more comfortable with reflective exercises or visual storytelling. Future research could compare photo-elicitation-based findings with those derived from traditional interviews to assess potential methodological differences.

Finally, as qualitative researchers, our positionality inevitably shapes the interpretation of findings. Our backgrounds in veterinary education and veterinary social work informed our engagement with student narratives and our focus on resilience, community, and professional identity. While this perspective aligns with contemporary discussions in veterinary education, it also presents an interpretive lens that should be acknowledged. Member checking was implemented to mitigate this influence, ensuring that participant perspectives remained central to the analysis.

### Future directions

Future research should aim to include larger, more demographically and experientially diverse samples to explore how factors such as cultural background, socioeconomic status, and personal life experiences shape veterinary students’ motivations and resilience strategies. Given that veterinary medicine has historically lacked racial and economic diversity (38), studies focusing on underrepresented populations could provide valuable insights into systemic barriers and support mechanisms within veterinary education.

Longitudinal research tracking students from their early training through their professional careers would offer deeper insight into how motivations evolve over time and how reflective practices contribute to long-term wellbeing and career satisfaction. This could help identify whether early engagement with intrinsic motivations mitigates burnout, improves retention, and enhances professional fulfillment.

Expanding the use of photo elicitation in veterinary education may also yield new insights into student wellbeing, identity development, and the impact of curriculum design. Future studies could explore its application in veterinary admissions, mentorship programs, and mental health initiatives to assess how reflective practices influence student engagement and resilience. Additionally, intervention-based research could test whether structured reflective exercises—such as guided photo elicitation workshops—enhance psychological endurance, ethical reasoning, and adaptability among veterinary students.

## Conclusion

Understanding the motivations of veterinary students is more than an academic exercise—it is a critical component of preparing the next generation of veterinarians to navigate the demands of a dynamic and challenging profession. This study’s findings, drawn from the integration of photo elicitation and thematic analysis, reveal motivations rooted in compassion, resilience, innovation, and service. These insights reflect the values that sustain students throughout their academic journeys and serve as a foundation for fostering professional fulfillment.

Veterinary education stands to benefit from integrating principles such as compassionate advocacy, spectrum of care, and community engagement into curricula. By emphasizing reflective practices, experiential learning, and mentorship, educational programs can cultivate resilience, address disparities in care, and prepare students for impactful careers. Moreover, these approaches align with broader trends in the profession, such as the increasing focus on wellbeing, job satisfaction, and ethical practice.

As veterinary medicine continues to evolve, so too must its educational frameworks. However, this does not mean that veterinary curricula should simply conform to student preferences; rather, it requires an intentional balance between fostering intrinsic motivation and maintaining the structured professional training necessary for veterinary practice. Research suggests that veterinary students often struggle with resilience and mental health, particularly in their early careers, contributing to high levels of attrition and professional dissatisfaction (1, 2). By integrating reflective exercises like photo elicitation into early veterinary training, educators can proactively engage students in self-exploration, reinforcing a sense of purpose that may act as a buffer against the inevitable challenges of the profession.

Our approach aligns with growing calls for veterinary education to address mental health preventatively rather than reactively. Students come to veterinary school to learn, but they also need to develop the psychological endurance necessary to sustain long careers in a demanding field. This requires acknowledging the tension between professional education—often framed as an authoritative transmission of expertise—and the reflective, contextual learning found in the humanities. While students must acquire technical competence under expert mentorship, fostering engagement with their own motivations and values is not a distraction but a means to reinforce professional identity and ethical decision-making.

This tension extends beyond veterinary education and into practice, particularly in the power dynamics of the exam room. Just as veterinary educators are positioned as authoritative experts in the classroom, veterinarians often assume a similar role with clients, which can inadvertently reinforce hierarchical and paternalistic approaches to care. This risks undermining client trust and adherence, particularly within the framework of spectrum of care, which relies on shared decision-making, transparency, and adaptability. Educational approaches that emphasize collaboration, self-reflection, and problem-solving—such as photo elicitation—not only challenge entrenched power structures in the classroom but also cultivate skills that translate into more effective, empathetic client communication. As Freire (25) argues, “Education must begin with the solution of the teacher-student contradiction, by reconciling the poles of the contradiction so that both are simultaneously teachers and students” (25, p. 72). Encouraging students to articulate their motivations, connect with their “why,” and integrate personal meaning into their training is not about catering to individual preferences—it is about preparing them to navigate the complexities of veterinary practice with humility and openness.

However, these shifts cannot be considered in isolation from the hidden curriculum—the implicit messages, values, and norms conveyed through institutional culture and informal interactions (38). Veterinary education has traditionally emphasized hierarchy, competitiveness, and rigid professional norms, sometimes at odds with explicit teachings on collaboration, wellbeing, and ethical practice. The hidden curriculum may subtly reinforce attitudes that discourage help-seeking behaviors, normalize stress as an unavoidable consequence of training, and prioritize technical expertise over communication and adaptability. Without intentional efforts to surface and critically examine these implicit messages, students may struggle to integrate the values of compassionate advocacy and shared decision-making into their professional identities. Reflective methodologies, such as photo elicitation, offer a means of making these underlying influences visible, allowing students to actively engage with and question the cultural forces shaping their educational experiences.

Until we start seeing veterinary students as collaborators in educational research, represented as such through participatory methodologies, and not merely subjects in a curriculum, we will struggle to evolve veterinary education to meet both student needs and societal demands. Addressing the influence of both explicit and hidden curricula is essential to creating an educational environment that prepares students not only for the technical demands of practice but also for the ethical and interpersonal complexities of veterinary medicine.

## Data Availability

The raw data supporting the conclusions of this article will be made available by the authors, without undue reservation.

## References

[1] Moffett JE Bartram DJ. Veterinary students’ perspectives on resilience and resilience-building strategies. J Vet Med Educ. (2017) 44:116–24. doi: 10.3138/jvme.0216-046R1, PMID: 28206832

[2] Sharma G Yukhymenko-Lescroart MA. Life purpose as a predictor of resilience and persistence in college students during the COVID-19 pandemic. J College Stu Ret Res Theor Prac. (2024) 26:334–54. doi: 10.1177/15210251221076828

[3] Cake MA McArthur MM Matthew SM Mansfield CF. Finding the balance: uncovering resilience in the veterinary literature. J Vet Med Educ. (2017) 44:95–105. doi: 10.3138/jvme.0116-025R, PMID: 28206842

[4] Gasper M. Resilience and compassion fatigue. Knoxville, TN: University of Tennessee (2023).

[5] Ward A May SA. The modern UK veterinary profession: photo-elicitation interviewing reveals that small animal and surgical images dominate. Vet Rec. (2019) 184:650–11. doi: 10.1136/vr.105046, PMID: 31023872

[6] Nett RJ Witte TK Holzbauer SM Elchos BL Campagnolo ER Musgrave KJ. Risk factors for suicide, attitudes toward mental illness, and practice-related stressors among US veterinarians. J Am Vet Med Assoc. (2015) 247:945–55. doi: 10.2460/javma.247.8.945, PMID: 26421408

[7] Bartram DJ Baldwin DS. Veterinary surgeons and suicide: a structured review of possible influences on increased risk. Vet Rec. (2010) 166:388–97. doi: 10.1136/vr.b4794, PMID: 20348468

[8] Zhang Y Hennebry-Leung M. A review of using photo-elicitation interviews in qualitative education research. Int J Qual Methods. (2023) 22:16094069231185456. doi: 10.1177/16094069231185456

[9] Harper D. Talking about pictures: a case for photo elicitation. Vis Stud. (2002) 17:13–26. doi: 10.1080/14725860220137345

[10] Torre D Murphy J. A different lens: using photo-elicitation interviews in education research. Educ Policy Anal Arch. (2015) 23:2051. doi: 10.14507/epaa.v23.2051, PMID: 35162190

[11] Bugos E Frasso R FitzGerald E True G Adachi-Mejia AM Cannuscio C. Practical guidance and ethical considerations for studies using photo-elicitation interviews. Prev Chronic Dis. (2014) 11:E189. doi: 10.5888/pcd11.140216, PMID: 25357257 PMC4215569

[12] Collier J. Photography in anthropology: a report on two experiments. Am Anthropol. (1957) 59:843–59. doi: 10.1525/aa.1957.59.5.02a00100

[13] Dworkin M Akintayo T Calem D Doran C Guth A Kamami EM. Life during the pandemic: an international photo-elicitation study with medical students. BMC Med Educ. (2021) 21:244. doi: 10.1186/s12909-021-02684-x, PMID: 33906671 PMC8078097

[14] Wilson R Varshney K Petrera M Hoff N Thiel V Frasso R. Reflections of graduating medical students: a photo-elicitation study. Med Sci Educ. (2023) 33:363. doi: 10.1007/s40670-023-01758-3, PMID: 36811080 PMC9933809

[15] Clark-Ibáñez M. Framing the social world with photo-elicitation interviews. Am Behav Sci. (2004) 47:1507–27. doi: 10.1177/0002764204266236

[16] Meo AI. Picturing students' habitus: the advantages and limitations of photo-elicitation interviewing in a qualitative study in the city of Buenos Aires. Int J Qual Methods. (2010) 9:149–71. doi: 10.1177/160940691000900203

[17] Small ML. “How many cases do I need?” on science and the logic of case selection in field-based research. Ethnography. (2009) 10:5–38. doi: 10.1177/1466138108099586

[18] Spillman L. Mixed methods and the logic of qualitative inference. Qual Sociol. (2014) 37:189–205. doi: 10.1007/s11133-014-9273-0

[19] American Veterinary Medical Association (AVMA). Job satisfaction rises for veterinarians. Schaumburg, IL: AVMA (2023).

[20] Desmond M. Relational ethnography. Theory Soc. (2014) 43:547–79. doi: 10.1007/s11186-014-9232-5

[21] Braun V Clarke V. Thematic analysis: A practical guide. London: SAGE Publications Ltd. (2022).

[22] Braun V Clarke V. Toward good practice in thematic analysis: avoiding common problems and be(com)ing a knowing researcher. Int J Transgender Health. (2023) 24:1–6. doi: 10.1080/26895269.2022.2129597, PMID: 36713144 PMC9879167

[23] McAdams DP. The stories we live by: Personal myths and the making of the self. New York, NY: William Morrow (1993).

[24] van Manen M. Beyond assumptions: shifting the limits of action research. Theory Pract. (1990) 29:152–7. doi: 10.1080/00405849009543448

[25] Freire P. Pedagogy of the oppressed. London: Routledge (1970).

[26] Brown CR Garrett LD Gilles WK Houlihan KE McCobb E Pailler S. Spectrum of care: more than treatment options. J Am Vet Med Assoc. (2021) 259:712–7. doi: 10.2460/javma.259.7.712, PMID: 34516261

[27] Masten AS. Ordinary magic: resilience processes in development. Am Psychol. (2001) 56:227–38. doi: 10.1037/0003-066X.56.3.227, PMID: 11315249

[28] Merck Animal Health. Merck animal health veterinary wellbeing study. Rahway, NJ: Merck Animal Health (2023).

[29] Fraser SW Greenhalgh T. Coping with complexity: educating for capability. BMJ. (2001) 323:799–803. doi: 10.1136/bmj.323.7316.799, PMID: 11588088 PMC1121342

[30] Epstein I Stevens B McKeever P Baruchel S. Photo elicitation interview (PEI): using photos to elicit children's perspectives. Int J Qual Methods. (2006) 5:1–11. doi: 10.1177/160940690600500301

[31] Bell MA Cake MA Mansfield CF. Beyond competence: why we should talk about employability in veterinary education. J Vet Med Educ. (2018 Spring) 45:27–37. doi: 10.3138/jvme.0616-103r1, PMID: 28657482

[32] Blackwell MJ. Bridging gaps in veterinary care: equity, access, and innovation. Vet Clin North Am Small Anim Pract. (2024) 54:169–79. doi: 10.1016/j.cvsm.2023.08.001, PMID: 39261112

[33] Evason MD Stein MR Stull JW. Impact of a Spectrum of care elective course on third-year veterinary Students' self-reported knowledge, attitudes, and competencies. J Vet Med Educ. (2023) 50:590–8. doi: 10.3138/jvme-2022-0010, PMID: 36112837

[34] Fingland RB Stone LR Read EK Moore RM. Preparing veterinary students for excellence in general practice: building confidence and competence by focusing on spectrum of care. J Am Vet Med Assoc. (2021) 259:463–70. doi: 10.2460/javma.259.5.463, PMID: 34388008

[35] Steffey EA Wolfe J Blackwell MJ. Addressing veterinarian mental health: strategies for sustainability. Front Vet Sci. (2023) 10:1184526. doi: 10.3389/fvets.2023.1184526, PMID: 37470072 PMC10352684

[36] Hafen Jr M Ratcliffe GC Rush BR. Veterinary medical student well-being: depression, stress, and personal relationships. J Vet Med Educ. (2013) 40:296–302. doi: 10.3138/jvme.1112-101R, PMID: 23975073

[37] Hafen Jr M Drake AS Elmore RG. Predictors of psychological well-being among veterinary medical students. J Vet Med Educ. (2023) 50:297–304. doi: 10.3138/jvme-2021-0133, PMID: 35587522

[38] Watson B Berliner E DeTar L McCobb E Frahm-Gilles W Henry E. Principles of veterinary community engagement. J Shelter Med Community Anim Health. (2024) 3:S2. doi: 10.56771/VCEprinciples.2024

[39] Whitcomb TL. Raising awareness of the hidden curriculum in veterinary medical education: a review and call for research. J Vet Med Educ. (2014) 41:344–9. doi: 10.3138/jvme.0314-032R1, PMID: 25335646

